# Admission Serum Calcium Level and Short-Term Mortality After Acute Ischemic Stroke: A Secondary Analysis Based on a Norwegian Retrospective Cohort

**DOI:** 10.3389/fneur.2022.889518

**Published:** 2022-06-15

**Authors:** Yuzhao Lu, Xin Ma, Kiarash Tazmini, Ming Yang, Xiaobing Zhou, Yang Wang

**Affiliations:** ^1^Department of Neurosurgery, The First Affiliated Hospital of Nanchang University, Nanchang University, Nanchang, China; ^2^Department of Thoracic Surgery, Jingshan Union Hospital of Huazhong University of Science and Technology, Wuhan, China; ^3^Department of Endocrinology, Morbid Obesity and Preventive Medicine, Faculty of Medicine, Oslo University Hospital, Oslo, Norway; ^4^Department of Neurosurgery, Central Theater General Hospital of Chinese PLA, Wuhan, China; ^5^Department of Neurosurgery, Beijing Chaoyang Hospital, Capital Medical University, Beijing, China

**Keywords:** serum calcium, albumin-corrected serum calcium, association, ischemic stroke, 30-day mortality, baseline

## Abstract

**Background:**

Disturbed serum calcium levels are related to the risk of stroke. However, previous studies exploring the correlation between serum calcium and the clinical outcome of ischemic stroke (IS) have shown inconsistent results.

**Object:**

The study aimed to investigate the relationship between admission serum calcium and 30-day mortality in patients with IS.

**Methods:**

A total of 876 IS patients from a Norwegian retrospective cohort were included for secondary analysis. The exposure variable and the primary outcome were albumin-corrected serum calcium (ACSC) at baseline and all-cause mortality within 30 days after the first admission, respectively. Multivariable logistic regression analysis was used to estimate the risk of 30-day mortality according to ACSC levels. Moreover, the potential presence of a non-linear relationship was evaluated using two-piecewise linear regression with a smoothing function and threshold level analysis. The stability of the results was evaluated by unadjusted and adjusted models.

**Results:**

The result of multiple regression analysis showed that ACSC at baseline was positively associated with the incidence of 30-day mortality after adjusting for the potential confounders (age, gender, serum glucose, hypertension, atrial fibrillation/atrial flutter, renal insufficiency, heart failure, chronic obstructive pulmonary disease, pneumonia, paralysis, and aphasia) (OR = 2.43, 95% CI 1.43–4.12). When ACSC was translated into a categorical variable, the ORs and 95% CIs in the second to the fourth quartile vs. the first quartile were 1.23 (0.56, 2.69), 1.16 (0.51, 2.65), and 2.13 (1.04, 4.38), respectively (*P* for trend = 0.03). Moreover, the results of two-piecewise linear regression and curve-fitting revealed a linear relationship between ACSC and 30-day mortality.

**Conclusion:**

ACSC is positively associated with 30-day mortality in IS patients, and the relationship between them is linear.

## Introduction

Stroke can cause a low quality of life for patients and their families, as well as a great burden and loss for society due to high rates of disability and mortality (1). Ischemic stroke (IS), which is the main subtype of stroke, accounts for about 60–80% (1) of all stroke cases according to the latest evidence. Given this, early risk stratification after acute IS may contribute to improving clinical decision-making.

Calcium is the most abundant mineral in the human body (2), widely taking part in various crucial physiological processes including signal transduction, maintenance of the stability of the cell membrane, coagulation process, movement of the smooth muscle or skeletal muscle, and endocrine function (3, 4). Serum calcium level in a normal physiological situation is strictly controlled to remain within a narrow range (5). Moreover, dyscalcemia has been demonstrated to be related to the risk of cardiovascular and cerebrovascular diseases (6–8).

To date, a limited amount of studies have addressed the association between serum calcium levels and IS outcomes, with conflicting results (9–13). In these studies, both low (10) and high levels (12) of serum calcium have been reported to correlate with poor outcomes of IS. One study reported a U shape association between serum calcium levels and in-hospital all-cause mortality (11). Furthermore, Asian and North American patients were the main subjects in previous studies, which failed to consider European populations. However, not only do the incidence rate and morbidity of stroke vary in different populations (14), in addition to the calcium metabolism (15).

Serum calcium is susceptible to serum albumin levels (16) and albumin-corrected serum calcium (ACSC) calculated according to the classic formula (17) is increasingly used in place of serum calcium in many clinical studies (10–13). Therefore, this study was designed to assess the correlation between ACSC and 30-day mortality in IS patients based on a Norwegian retrospective cohort.

## Methods

### Data Source

Initial data were downloaded from the public database “DRYAD” (www.datadryad.org). In this database, Tazmini et al. (18) authorized the use of their data in the DRYAD database. Thus, this secondary research based on the raw data for a different research hypothesis was permitted. In addition, the original corresponding author, Kiarash Tazmini, was listed as a co-author with their consent for the contribution of their team in data collection and making their data publicly available.

**Figure 1:**
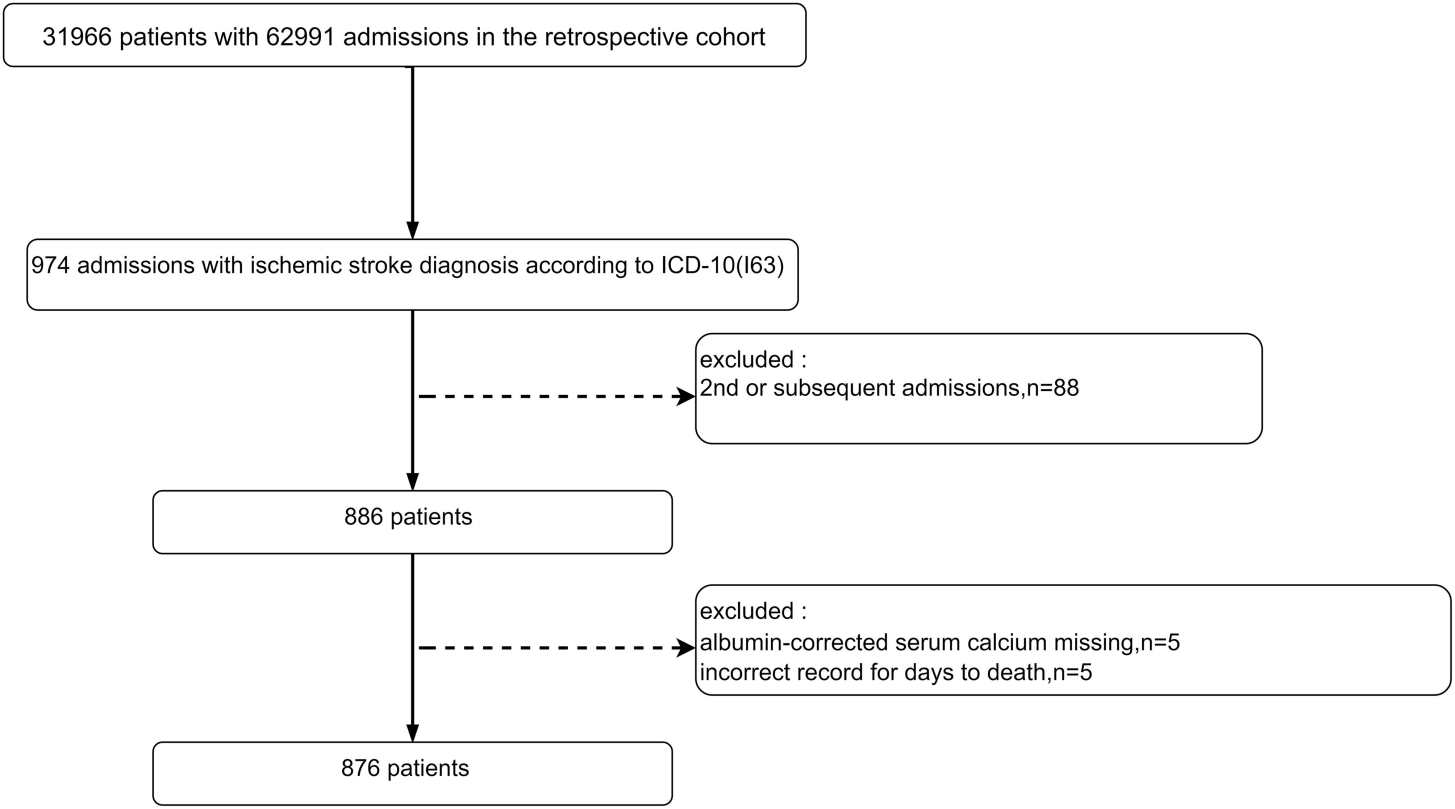
Flowchart of screening the study population.

The original research was a single-center retrospective cohort study that included 31,966 unique patients (62,991 registered admission information) who visited the emergency department of the Diakonhjemmet Hospital in Oslo (Norway) from 2010 to 2015. In this study, a total of 974 visits (admission information) with a principal diagnosis of IS (ICD-10, I63) were selected from the raw cohort according to the International Classification of Diseases 10th revision (ICD-10). The raw data included information on multiple hospitalizations for the same patient, but only the first one for each patient was considered in this study. A total of 886 unique IS patients were identified according to their first admission information after excluding the second or subsequent admissions. Subsequently, 10 patients were excluded for missing ACSC information (*n* = 5) or wrong death information (*n* = 5). Ultimately, 876 IS patients were included (Figure 1).

The original research was approved by the Norwegian Regional Committee for Medical and Health Research Ethics South East as a quality study, which did not require ethical approval (19). Informed consent was also not necessary because all the data were anonymously processed (19). Thus, separate ethical approval was not required for this secondary analysis. Finally, this study complied with the Helsinki Declaration.

All the laboratory indicators were taken from the first laboratory results after admission. Serum calcium (mmol/L), serum-albumin (g/L), serum-sodium (mmol/L), serum-potassium (mmol/L), serum-glucose (mmol/L), serum-phosphate (mmol/L), and serum-magnesium (mmol/L) were recorded in the original data.

The ACSC levels in the previous study were calculated according to a standard formula and the epidemiological data of the northern European population (20). And the calculation formula is as follows: ACSC = measured serum-calcium level + 0.020 × (41.3–serum-albumin) where 41.3 g/L is the albumin median (19). The unit of ACSC and serum calcium was converted to mg/dl.

### Co-morbidities

Secondary diagnostic information was used to identify co-morbidities including diabetes (ICD-10: E10-E14), hypertension (ICD-10: I10), hyperlipemia (ICD-8: E78), atrial fibrillation/atrial flutter (ICD-10: I48), heart failure (ICD-10: I50), renal insufficiency (ICD-10: N18), chronic obstructive pulmonary disease (ICD-10: J42–44), coronary heart disease (ICD-10: I25), chronic obstructive pulmonary disease (ICD-10: J42–44), cancer (ICD-10: C0-C9, Z51.0-3), malnutrition (ICD-10: E40-E46), and pneumonia (ICD-10: J98, J69, J11-18). Moreover, common complications associated with strokes such as paralysis (ICD-10: G80-G83), epilepsy (ICD-10: G40), cognitive disorder (ICD-10: F06), and aphasia (ICD-10: F80, R47) were also considered. All the above-mentioned co-morbidities were processed into categorical variables to facilitate the statistical analysis.

### Outcome

The primary outcome was all-cause mortality within 30 days after the first admission.

### Missing Data

The missing data of covariates of all 876 patients, in the final analysis, are shown in Table 1. Serum-phosphate and serum-magnesium were missing a large portion of information and were thus converted into categorical variables to address the lower statistical power and potential bias caused by excluding missing data. Dummy variables were used to identify the missing values of the covariate (21).

**Table 1:** Baseline characteristics of the patients according to quartiles of ACSC level.

**ACSC (mg/dl)**	**Quantile1**	**Quantile 2**	**Quantile 3**	**Quantile 4**	***P*** **-value**
*N* = 876	209	223	206	238	
Age (years)	75.09 ± 13.53	76.91 ± 12.61	76.77 ± 11.83	79.91 ± 10.61	0.002
Gender (male), *n* (%)	119 (56.94%)	108 (48.43%)	95 (46.12%)	75 (31.51%)	<0.001
Serum-sodium (mmol/L, mean ± SD) (1 missing)	139.19 ± 3.99	139.66 ± 3.23	139.59 ± 3.01	139.26 ± 3.89	0.6
Serum-glucose (mmol/L, mean ± SD)	6.82 ± 2.33	6.77 ± 2.09	6.85 ± 2.12	7.19 ± 2.32	0.01
Serum-potassium (mmol/L) (3 missing)	4.11 ± 0.44	4.14 ± 0.43	4.12 ± 0.40	4.17 ± 0.45	0.4
Serum-phosphate (mmol/L) tertiles, *n* (%)					0.1
Tertile 1 (≤ 0.97)	25 (11.96%)	18 (8.07%)	28 (13.59%)	28 (11.76%)	
Tertile 2 (0.98–1.12)	20 (9.57%)	24 (10.76%)	26 (12.62%)	24 (10.08%)	
Tertile 3 (≥1.13)	22 (10.53%)	20 (8.97%)	29 (14.08%)	40 (16.81%)	
Not recorded	142 (67.94%)	161 (72.20%)	123 (59.71%)	146 (61.34%)	
Serum-magnesium (mmol/L) tertiles, *n* (%)					0.02
Tertile 1 (≤ 0.78)	23 (11.00%)	11 (4.93%)	29 (14.08%)	39 (16.39%)	
Tertile 1 (0.79–0.84)	22 (10.53%)	28 (12.56%)	25 (12.14%)	23 (9.66%)	
Tertile 1 (≥0.85)	28 (13.40%)	24 (10.76%)	29 (14.08%)	32 (13.45%)	
Not recorded	136 (65.07%)	160 (71.75%)	123 (59.71%)	144 (60.50%)	
Diabetes, *n* (%)	15 (7.18%)	19 (8.52%)	21 (10.19%)	20 (8.40%)	0.7
Hyperlipemia, *n* (%)	3 (1.44%)	4 (1.79%)	12 (5.83%)	15 (6.30%)	0.008
Hypertension, *n* (%)	52 (24.88%)	56 (25.11%)	61 (29.61%)	71 (29.83%)	0.5
Atrial fibrillation/atrial flutter, *n* (%)	56 (26.79%)	57 (25.56%)	50 (24.27%)	67 (28.15%)	0.8
Heart failure, *n* (%)	4 (1.91%)	7 (3.14%)	7 (3.40%)	8 (3.36%)	0.8
Renal insufficiency, *n* (%)	6 (2.87%)	11 (4.93%)	10 (4.85%)	13 (5.46%)	0.6
COPD, *n* (%)	1 (0.48%)	6 (2.69%)	4 (1.94%)	1 (0.42%)	0.1
CHD, *n* (%)	11 (5.26%)	11 (4.93%)	14 (6.80%)	9 (3.78%)	0.6
Cancer, *n* (%)	3 (1.44%)	4 (1.79%)	3 (1.46%)	7 (2.94%)	0.6
Malnutrition, *n* (%)	6 (2.87%)	3 (1.35%)	1 (0.49%)	9 (3.78%)	0.08
Dehydration, *n* (%)	9 (4.31%)	9 (4.04%)	7 (3.40%)	9 (3.78%)	1.0
Pneumonia, *n* (%)	6 (2.87%)	12 (5.38%)	9 (4.37%)	13 (5.46%)	0.5
Paralysis, *n* (%)	15 (7.18%)	24 (10.76%)	16 (7.77%)	31 (13.03%)	0.1
Epilepsy, *n* (%)	4 (1.91%)	0 (0.00%)	2 (0.97%)	2 (0.84%)	0.2
Cognitive disorder, *n* (%)	3 (1.44%)	8 (3.59%)	4 (1.94%)	13 (5.46%)	0.07
Aphasia, *n* (%)	18 (8.61%)	17 (7.62%)	9 (4.37%)	19 (7.98%)	0.3
30-day mortality	13 (6.22%)	20 (8.97%)	16 (7.77%)	36 (15.13%)	0.008

*All data in the ACSC subgroups are expressed as mean ± SD or number (%). ACSC: quantile 1: ≤ 9.26 mg/dl; quantile 2: 9.30–9.50 mg/dl; quantile 3: 9.54–9.74 mg/dl, and quantile 4: ≥9.78 mg/dl. ACSC, albumin-corrected serum calcium; COPD, chronic obstructive pulmonary disease; CHD, coronary heart disease*.

Multiple imputations based on five replications and a chained equation approach in the R MI procedure were used to deal with missing data (22). Moreover, a comparison between primary data and interpolation data was performed for the sensitivity analysis. The result is shown in Supplementary Figure 2 and Supplementary Table 1.

### Statistical Analysis

Statistical analysis was performed using EmpowerStats (www.empowerstats.com, X&Y Solutions, Inc., Boston, MA) and the statistical software package R (http://www.R-project.org, The R Foundation).

Mean ± standard or median (interquartile range) were used to describe continuous variables and categorical variables were expressed as percentages. One-way ANOVA test for continuous variables with normal distribution, Kruskal–Wallis *H*-test for continuous variables with skewed distribution, and chi-square tests (or Fisher's exact test) for categorical variables were used to analyze differences between or among groups.

Multiple logistic regression analysis was used to assess the specific relationships between the exposure (ACSC) and outcome (30-day mortality); odds ratio (OR) and 95% confidence interval (CI) were used to evaluate the risk.

Four models were built to control for the effect of confounding factors: (1) crude model, i.e., unadjusted. (2) Model I, which was adjusted for age and gender. (3) Model II, which was adjusted for age, gender, serum glucose, atrial fibrillation/atrial flutter, renal insufficiency, heart failure, chronic obstructive pulmonary disease, cancer, pneumonia, paralysis, and aphasia. Covariates were included as potential confounders in the final models if they changed the estimates of admission albumin-corrected serum calcium on 30-day mortality by more than 10% or were significantly associated with 30-day mortality (23); gender as a basic variable was also included in the fully adjusted model. (4) Model III, which was additionally adjusted for serum-phosphate tertiles, serum-magnesium tertiles, cognitive disorder, and epilepsy based on Model II (In Model III, the potential influence of missing variables and clinical significance of the other two neurological complications were considered).

A two-piecewise linear regression model and curve fitting were used to examine the potential linear relationship and threshold effect.

### Sensitivity Analysis

ACSC quartiles were also used to test the stability of multiple regression results, and the linear tests were performed by assigning medians to each ACSC quartile as a continuous variable in the models (24).

An *E*-value was used to explore the potential of unmeasured confounding between ACSC and 30-day mortality. The *E*-value was defined as the required magnitude for an unmeasured confounder to overturn the observed association between ACSC and 30-day mortality (25).

## Results

### Baseline Characteristics of Participants

The average age of participants was 77.26 ± 12.26 (34–100) years and 54.68% were female. The baseline characteristics and co-morbidities of participants are listed in Table 1 by ACSC quartiles. Age, gender, serum-glucose, serum-magnesium (tertiles), hyperlipemia, and 30-day mortality of the ACSC quartile groups were statistically different (all *p* < 0.05).

### Univariate Analysis Related to 30-Day Mortality

The outcome of 30-day mortality was chosen as a dependent variable, and univariate analysis was used to investigate which independent variable was related to 30-day mortality. The results indicated that age (OR = 1.08, 95% CI 1.05–1.11), ACSC (OR = 2.38, 95% CI 1.50–3.78), serum-glucose (OR = 1.11, 95% CI 1.03–1.21), hypertension (yes vs. no: OR = 0.54, 95% CI 0.30–0.97), heart failure (yes vs. no: OR = 6.46, 95% CI 2.83–14.74), renal insufficiency (yes vs. no: OR = 3.91, 95% CI 1.87–8.14), pneumonia (yes vs. no: OR = 7.41, 95% CI 3.76–14.61) and aphasia (yes vs. no: OR = 2.39, 95% CI 1.22–4.68) were all associated with 30-day mortality (Table 2).

**Table 2:** Univariate analysis related to 30-day mortality.

**Variables**	**Statistics**	**OR (95% CI)**	***p-*** **value**
Age (year)	77.26 ± 12.26	1.08 (1.05, 1.11)	<0.0001
**Gender**			
Male	397 (45.32%)	Reference	
Female	479 (54.68%)	1.34 (0.85, 2.12)	0.2
ACSC (mg/dL)	9.56 ± 0.43	2.38 (1.50, 3.78)	0.0002
Serum-sodium (mmol/L)	139.42 ± 3.56	1.05 (0.98, 1.12)	0.2
Serum-glucose (mmol/L)	6.91 ± 2.22	1.11 (1.03, 1.21)	0.01
Serum-potassium (mmol/L)	4.13 ± 0.43	1.40 (0.84, 2.32)	0.2
**Serum-phosphate (mmol/L) tertiles**, ***n*** **(%)**			
Tertile 1 (≤ 0.97)	99 (11.30%)	Reference	
Tertile 2 (0.98–1.12)	94 (10.73%)	1.60 (0.65, 3.95)	0.3
Tertile 3 (≥1.13)	111 (12.67%)	1.44 (0.60, 3.50)	0.4
Not recorded	572 (65.30%)	0.94 (0.44, 1.97)	0.9
**Serum-magnesium (mmol/L) tertiles**, ***n*** **(%)**			
Tertile 1 (≤ 0.78)	102 (11.64%)	Reference	
Tertile 2 (0.79–0.84)	98 (11.19%)	1.05 (0.41, 2.63)	0.9
Tertile 3 (≥0.85)	113 (12.90%)	1.63 (0.71, 3.74)	0.3
Not recorded	563 (64.27%)	0.86 (0.42, 1.76)	0.7
**Diabetes**, ***n*** **(%)**			
No	801 (91.44%)	Reference	
Yes	75 (8.56%)	0.79 (0.33, 1.89)	0.6
**Hyperlipemia**, ***n*** **(%)**			
No	842 (96.12%)	Reference	
Yes	34 (3.88%)	-	§
**Hypertension**, ***n*** **(%)**			
No	637 (72.72%)	Reference	
Yes	239 (27.28%)	0.54 (0.30, 0.97)	0.04
**Atrial fibrillation/atrial flutter**, ***n*** **(%)**			
No	646 (73.74%)	Reference	
Yes	230 (26.26%)	1.52 (0.94, 2.45)	0.08
**Heart failure**, ***n*** **(%)**			
No	850 (97.03%)	Reference	
Yes	26 (2.97%)	6.46 (2.83, 14.74)	<0.0001
**Renal insufficiency**, ***n*** **(%)**			
No	835 (95.32%)	Reference	
Yes	40 (4.57%)	3.91 (1.87, 8.14)	0.0003
**Chronic obstructive pulmonary disease (%)**			
No	864 (98.63%)	Reference	
Yes	12 (1.37%)	3.18 (0.84, 11.98)	0.09
**Coronary heart disease**, ***n*** **(%)**			
No	831 (94.86%)	Reference	
Yes	45 (5.14%)	1.17 (0.45, 3.06)	0.7
**Cancer**, ***n*** **(%)**			
No	859 (98.06%)	Reference	
Yes	17 (1.94%)	2.03 (0.57, 7.21)	0.3
**Malnutrition**, ***n*** **(%)**			
No	857 (97.83%)	Reference	
Yes	19 (2.17%)	1.10 (0.25, 4.83)	0.9
**Dehydration**, ***n*** **(%)**			
No	842 (96.12%)	Reference	
Yes	34 (3.88%)	0.90 (0.27, 3.00)	0.9
**Pneumonia**, ***n*** **(%)**			
No	836 (95.66%)	Reference	
Yes	40 (4.57%)	7.41 (3.76, 14.61)	<0.0001
**Paralysis**, ***n*** **(%)**			
No	790 (90.18%)	Reference	
Yes	86 (9.82%)	1.78 (0.94, 3.36)	0.08
**Epilepsy**, ***n*** **(%)**			
No	868 (99.09%)	Reference	
Yes	8 (0.91%)	-	§
**Cognitive disorder**, ***n*** **(%)**			
No	848 (96.80%)	Reference	
Yes	28 (3.20%)	0.34 (0.05, 2.51)	0.3
**Aphasia**, ***n*** **(%)**			
No	813 (92.81%)	Reference	
Yes	63 (7.19%)	2.39 (1.22, 4.68)	0.01

*Data are expressed as OR (95% CI) p-value. ACSC, albumin-corrected serum calcium*.

*^§^The model failed because of the small sample size*.

### Multivariate Logistic Regression Analysis of ACSC and 30-Day Mortality

ACSC was chosen as the independent variable and 30-day mortality as the dependent variable in the multiple regression equation. Other variables were used as covariates to adjust the model to prove the stability of the results, and four models were built. No covariates were adjusted in the crude model and the result showed that ACSC was independently associated with 30-day mortality (OR = 2.38, 95% CI 1.50–3.78). The result in the Model I also revealed that ACSC was independently related to 30-day mortality (OR = 2.52, 95% CI 1.52–4.18) after adjusting for age and gender. Moreover, the result was similar in the Model II adjusted for covariates such as age, gender, serum glucose, hypertension, atrial fibrillation/atrial flutter, renal insufficiency, heart failure, chronic obstructive pulmonary disease, pneumonia, paralysis, and aphasia (OR = 2.43, 95% CI 1.43–4.12). In Model III which additionally adjusted for serum-phosphate tertiles, serum-magnesium tertiles, cognitive disorder, and epilepsy based on Model II, the result was also robust (OR = 2.77, 95% CI 1.59–4.84) (Table 3).

**Table 3:** Multivariate regression analysis of ACSC and 30-day mortality.

**Exposure**	**Crude model**	**Mode I**	**Mode II**	**Model III**
ACSC (continuous)	2.38 (1.50, 3.78)	2.52 (1.52, 4.18)	2.43 (1.43, 4.12)	2.77 (1.59, 4.84)
**ACSC quartiles**				
Quantile 1	Reference	Reference	Reference	Reference
Quantile 2	1.49 (0.72, 3.07)	1.38 (0.66, 2.90)	1.23 (0.56, 2.69)	1.22 (0.56, 2.69)
Quantile 3	1.27 (0.59, 2.71)	1.25 (0.57, 2.70)	1.16 (0.51, 2.65)	1.17 (0.51, 2.67)
Quantile 4	2.69 (1.38, 5.22)	2.32 (1.17, 4.63)	2.13 (1.04, 4.38)	2.21 (1.07, 4.56)
*P* for trend	0.002	0.01	0.03	0.02

*Data are expressed as OR (95% CI) p-value. ACSC, albumin-corrected serum calcium (mg/dl); ACSC, quantile 1: ≤ 9.26 mg/dl; quantile 2: 9.30–9.50 mg/dl; Quantile 3: 9.54–9.74 mg/dl, and quantile 4: ≥9.78 mg/dl*.

*Crude model: not adjusted*.

*Mode I: adjusted for age and gender*.

*Mode II: adjusted for age, gender, serum glucose, atrial fibrillation/atrial flutter, renal insufficiency, heart failure, chronic obstructive pulmonary disease, cancer, pneumonia, paralysis, and aphasia*.

*Model III: adjusted for age, gender, serum glucose, serum-phosphate tertiles, serum-magnesium tertiles, atrial fibrillation/atrial flutter, renal insufficiency, heart failure, chronic obstructive pulmonary disease, cancer, pneumonia, paralysis, aphasia, cognitive disorder, and epilepsy*.

### Sensitivity Analysis

The results of the linear trend tests of the four models all showed that higher ACSC quartile groups were significantly related to an increased risk of 30-day mortality. And the *p*-values for the trends in the crude model, Model I, Model II, and Model III were 0.002, 0.01, 0.03, and 0.02, respectively (Table 3).

An *E*-value was calculated to assess the sensitivity to unmeasured confounding. The primary findings were stable unless an unmeasured confounder existed and was highly positively related to ACSC (OR ≥ 4.29) and 30 day-mortality (OR ≥ 4.29).

### Curve Fitting and Two-Piecewise Linear Regression Model of ACSC and 30-Day Mortality

**Figure 2:**
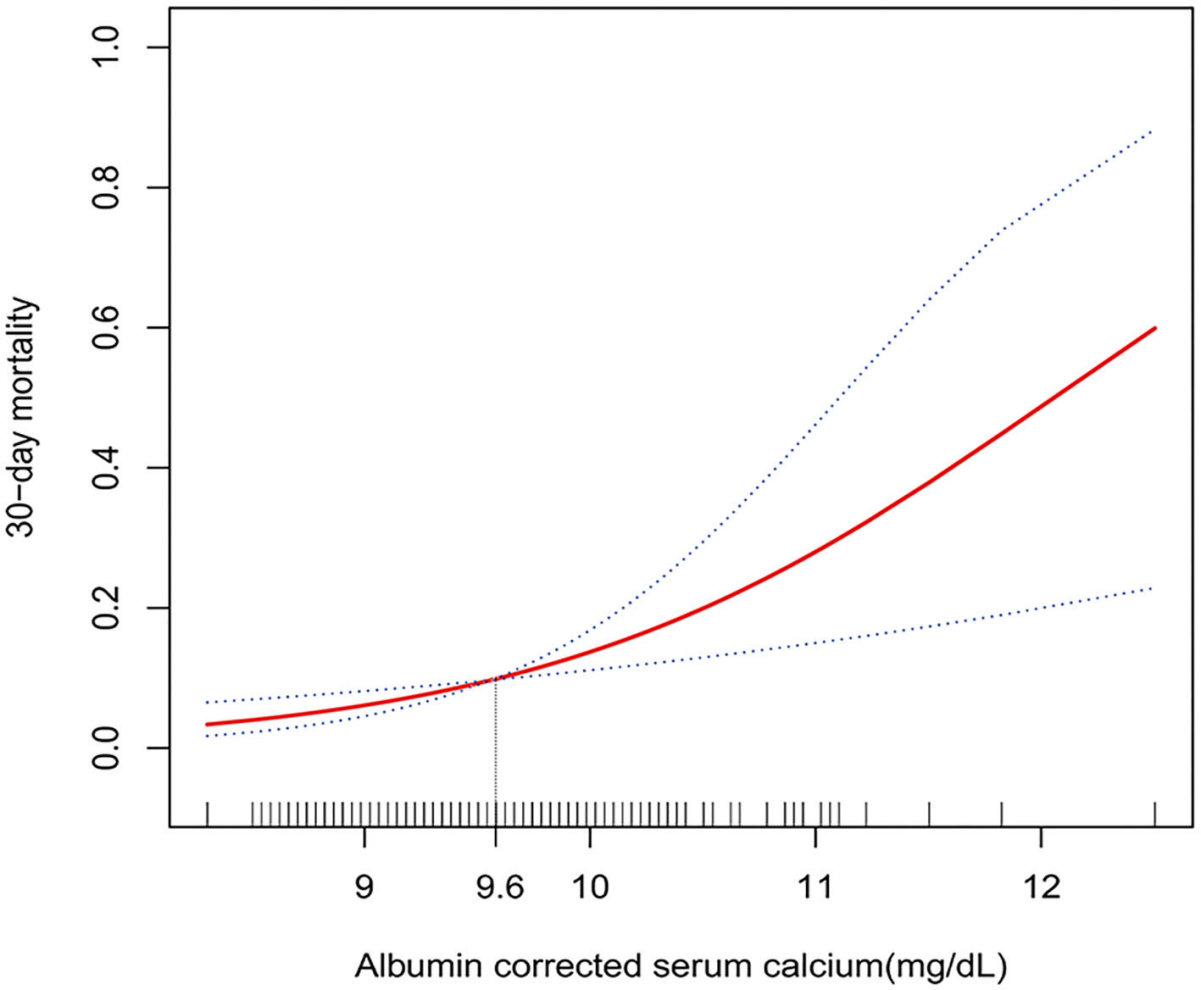
Multivariate adjusted smooth curve-fitting for association between ACSC and 30-day mortality. Same confounder factors as in model III: age, gender, serum glucose, serum-phosphate tertiles, serum-magnesium tertiles, atrial fibrillation/atrial flutter, renal insufficiency, heart failure, chronic obstructive pulmonary disease, cancer, pneumonia, paralysis, aphasia, cognitive disorder, and epilepsy, were adjusted. The red line represents the best-fit line, and the blue lines are 95% CIs. The potential demarcation point was 9.6 mg/dl according to the smoothing spline plots.

Curve fitting and two-piecewise linear regression analysis were used to investigate a potential non-linear association between ACSC and 30-day mortality. The result of curve fitting adjusted for age, gender, serum glucose, hypertension, atrial fibrillation/atrial flutter, renal insufficiency, heart failure, chronic obstructive pulmonary disease, pneumonia, cognitive disorder, epilepsy, paralysis, and aphasia showed a curve that continued to rise (Figure 2), and the results for the other three models were the same (Supplementary Figure 1). Furthermore, despite two different effective sizes based on the demarcation point (9.6 mg/dl) of the curve fitting was observed in the two-piecewise linear regression analysis adjusted according to Model II, the *p*-value for the likelihood ratio test was 0.7 (Table 4).

**Table 4:** Two-piecewise linear regression analysis for ACSC and 30-day mortality.

**ACSC (mg/dL)**	**Crude model**	**Model I**	**Model II**	**Model III**
<9.6	2.97 (0.85, 10.35)	2.21 (0.62, 7.88)	1.90 (0.51, 7.16)	2.03 (0.52, 7.83)
≥9.6	2.18 (1.14, 4.17)	2.63 (1.30, 5.31)	2.62 (1.26, 5.47)	3.17 (1.47, 6.84)
*P*-value for likelihood ratio test	0.7	0.8	0.7	0.6

*Data are expressed as OR (95% CI) p-value, ACSC, albumin-corrected serum calcium (mg/dl)*.

*Crude model: not adjusted*.

*Model I: adjusted for age and gender*.

*Model II: adjusted for age, gender, serum glucose, atrial fibrillation/atrial flutter, renal insufficiency, heart failure, chronic obstructive pulmonary disease, cancer, pneumonia, paralysis, and aphasia*.

*Model III: adjusted for age, gender, serum glucose, serum-phosphate tertiles, serum-magnesium tertiles, atrial fibrillation/atrial flutter, renal insufficiency, heart failure, chronic obstructive pulmonary disease, cancer, pneumonia, paralysis, aphasia, cognitive disorder, and epilepsy*.

The other three models showed similar results and the *p*-values for the likelihood ratio test were all >0.05 (Table 4). Thus, no threshold effect was observed according to the demarcation point, indicating that the relationship between ACSC and 30-day mortality was linear.

## Discussion

This study revealed an appreciable positive association between ACSC and 30-day mortality in IS patients. The relevant results were also significant after adjustment of the three different models with potential confounding factors. ACSC was then translated into a categorical variable (quartile) to analyze the sensitivity of the results, and the results were stable (OR values gradually increased significantly, from the second to the fourth quartile, and the values of p for trend were <0.05 in the four models). Furthermore, the results of curve fitting and the two-piecewise linear regression model revealed a stable linear relationship.

Despite the pivotal role of calcium ions in neuronal damage after ischemic events having been demonstrated as early as 1998 (26), few studies have focused on the correlation between baseline serum calcium and the clinical outcome in patients with IS. In a retrospective cohort study involving 173 IS patients, Buck et al. (9) explored between admission serum calcium and infarct volumes on diffusion-weighted imaging and their results revealed that high serum calcium levels were related to small cerebral infarct area and good outcomes. The study of Buck et al. (9) adjusted for serum glucose, blood pressure, co-morbidities, and stroke subtype in the final model, but serum albumin which might affect the serum calcium level was not considered. However, another study based on the Virtual International Stroke Trials Archive demonstrated that elevated 72–96-h serum calcium levels are related to 3-month functional outcome, but earlier (<4.5 h) serum calcium is not associated with the functional outcome (10). The result revealed that serum calcium levels can reflect a secondary epiphenomenon of stroke. However, the variation of intra-individual calcium levels, in reality, is ~2% (27), and the transportation of extracellular into neuronal cells would not significantly alter serum calcium levels. In 2010, an Israeli clinical study including 784 patients revealed that too high and too low serum calcium levels were both correlated with long-term mortality in female stroke patients (11). Moreover, a Korean cohort study that included 1915 IS patients showed that elevated admission ACSC levels are related to a short-time functional outcome and long-term mortality in IS patients (12). Admission serum calcium and ACSC were both included in the analysis, and the results of ACSC showed a significantly increased risk of all-cause death with the ACSC level elevating, but there were no positive results for serum calcium. Given the physiologic characteristic that more than 50% of calcium ions are combined with albumin and the difficulty for the measurement of ionized calcium in clinical practice, ACSC might be a better parameter than serum calcium to assess the effect of calcium. Most recently, a China national stroke cohort study demonstrated a high risk of long-term mortality of IS patients in the top quartile group of ACSC levels, with no statistical difference between patients of different genders (13).

Heterogeneity between the various study populations may have led to the inconsistency in the findings. Epidemiologic evidence showed that the incidence and mortality of stroke differed in various populations (14), in addition to calcium metabolism in different ethnicity (15). It was noteworthy that the relationship between serum calcium level and risk of stroke in Swedish and Korean populations also showed opposite results (7, 8). This study added evidence of the correlation between ACSC and clinical outcomes in IS patients from a different populations. Compared with other countries at a similar economical level, Nordic countries were with cold climate (28), short sunshine time (29), and plant-based diet pattern (30) which may impact the vitamin-D intake and serum calcium level. However, due to the nature of a retrospective study, the ethnicity, diet, climate, and environment of the selected sample group could not be included in the analysis. Prospective, large sample size studies that included these population characteristics are required in the future to accurately explore the risk range of serum calcium for Norwegian IS patients. In addition, due to the raw data of this study are from a large emergency cohort, ACSC may have a broad application prospect for IS patients admitted to emergency departments especially in primary or smaller Emergency Departments as a brief blood biomarker that could be quickly obtained at admission. Moreover, the causality of admission serum calcium level and short-term mortality could not be determined in this study. If future studies can demonstrate that admission elevated serum calcium cause increased mortality, clinical treatment including intravenous rehydration, enhancing kidney clearance of calcium (loop diuretics, calcitonin, and haemodialysis), calcium channel blocker, and limiting calcium and Vitamin D supplementation, might be beneficial to improve ischemic stroke patient outcomes.

The results of this study were compatible with the results of the previous study on the Chinese cohort (13). This study revealed that higher baseline ACSC levels were associated with 30-day mortality in IS patients. After processing ACSC as a categorical variable (quartiles), our results demonstrated that the top quartile group of ACSC levels most significantly increased the 30-day mortality. This result was also in agreement with the recent study from China (13), whose outcome is 1-year mortality (top quartile group, HR = 1.56, fully adjusted model). Moreover, previous studies had also demonstrated potential correlations between low serum calcium levels and cerebral infarct volume and short-term functional outcome, and U-shape relationship between baseline serum calcium and long-term mortality were shown by Appel et.al. Thus, we used curve fitting and the two-piecewise linear regression analyses to more accurately explore the relationship in this study. The results illustrated a linear relationship without threshold effect between ACSC level and 30-day mortality in IS patients, unlike the non-linear result of a previous study from the study of Appel et al. (11). In addition to differences in study populations, the study by Appel et al. (11) included both ischemic and hemorrhagic stroke patients, and different mediating mechanisms between serum calcium and mortality may account for their results, but this possibility needs to be verified by further studies.

Although extant studies demonstrated that high serum calcium levels were significantly associated with the risk of stroke (7) and clinical outcomes (12, 13), the pathophysiological mechanisms remain undefined. Previous studies revealed the key role of serum calcium in the promotion of vascular calcification, which is a complex process including the promotion of osteogenic/chondrogenic differentiation, vesicle release, cell apoptosis, loss of inhibitors, and extracellular matrix degradation (31, 32), leading to atherosclerosis. Thus, elevated serum calcium levels may accelerate the process of atherosclerosis, cardio-cerebrovascular calcification, and plaque rupture which has been associated with poor clinical outcomes (33–35). In addition, calcium ion is a crucial intracellular messenger, and plays a key role in neuronal damage and cell death (26). Furthermore, mitochondrial damage caused by high calcium concentration may be another mechanism (36). Moreover, recent research has shown that calcium ions could affect the cortical spreading depolarization after ischemic injury by regulating microglia activity (37). The association between elevated extracellular serum calcium levels and microglial calcium overload leading to cortical spreading depolarizations may explain the relationship between serum calcium levels and poor IS outcome. However, the inconsistent results of different studies suggested that there were more complicated mechanisms are needed to explain the effects of different ranges of serum calcium on all-cause mortality for IS patients, and the specific mechanisms need to be further studied.

This study has several advantages. First, the results of univariate analysis, regression coefficient change, and previous literature were used to select the covariates. Second, curve fitting and two-piecewise linear regression were used to explore a potential non-linear relationship, as shown in a previous study. Third, one crude model and four models adjusted with potential variables were used to test the stability of the results. Finally, ACSC was taken as a continuous variable and categorical variable into the multiple regression equation to avoid the contingency of the analysis, and the sensitivity analysis and trend test were used.

However, the following limitations exist. First, owing to the retrospective nature of this study, the non-inclusion of patients with missing ACSC information or wrong death information would lead to selection bias. Thus, the baseline information between the included group and the excluded group was compared, and the results showed no significant differences between the groups (Supplementary Table 2). Second, even though the ACSC level was calculated according to the standard formula, since the relationship between serum albumin and serum calcium could be more complex in the disease state, in addition to some studies having underlined that this formula could overestimate calcium levels (38, 39), further studies are needed to explore the actual relationship among serum calcium, serum albumin, and 30-day mortality. Though first-time laboratory results at admission, which are more likely to reflect the initial state of the patient at the onset, were used, it would be better to examine the dynamic changes in ACSC in future studies to understand the potential mechanism of the associations. Because of the retrospective study design, we could not confirm the time of blood collection, which will influence the ACSC level. Thus, further prospective studies with predesigned identical examination times are required. Third, the presence of unmeasured confounders could not be excluded. Since the secondary analysis originated from a retrospective cohort, variables that were not collected could not be adjusted. *E*-value was used to explore the potential for unmeasured confounding between ACSC and 30-day mortality and the result showed that an unmeasured confounder was unlikely to explain the entirety of the mortality effect. Pre-stroke medications such as calcium and vitamin D supplements might affect the level of admission to ACSC, and patients with chronic comorbidities were more likely to have a medication history. A stratification analysis was performed on comorbidity and non-comorbidity subgroups, revealing that the results remained stable in both subgroups. This means that the results remain stable even in the non-comorbidity patients who may be less likely to take medication before stroke (Supplementary Table 3). In addition, given that the medication treatment in the hospital would tend to a bias toward the null, it is postulated that the unmeasured confounding of medication treatment in the hospital might underestimate the observed effect. Neurological function (NIHSS score or Norwegian trial Scandinavian Stroke Scale), IS subtypes, and functional status before stroke are important information to evaluate the outcome of stroke patients. Therefore, common complications associated with neurological function including paralysis, epilepsy, cognitive disorder, and aphasia were additionally adjusted, and the results were also robust after adjustment (Model III). Moreover, some studies have revealed a negative association between serum calcium at the baseline and admission neurological function (9, 40). Therefore, adjustment for the neurological function tends to elevate the estimated effect. Finally, the participants in this study were represented by the Norwegian population, and the findings could not necessarily apply to other populations.

## Conclusions

Admission albumin-corrected serum calcium in ischemic stroke patients was positively correlated with 30-day mortality, and the relationship between them was almost linear.

## Data Availability Statement

The datasets used in this study are publicly available and can be accessed via the database www.Datadryad.org.

## Ethics Statement

The original research was approved by the Norwegian Regional Committee for Medical and Health Research Ethics South East as a quality study, which did not need ethical approval. Thus, our secondary analysis based on this original research did not require separate ethical approval. This research was performed according to the Declaration of Helsinki. All data was processed anonymously and therefore written informed consent from the patients/participants was not required for this study in accordance with the national legislation and the institutional requirements.

## Author Contributions

Conceptualization and methodology: YL and XM. Data source: KT. Software and writing—original draft preparation: YL. Visualization: XM. Writing—reviewing and editing: YW, MY, and XZ. All authors listed have read and approved the manuscript.

## Funding

This study was supported by the National Natural Science Foundation of China (No. 81960330) (http://www.nsfc.gov.cn) and Jiangxi Provincial Department of Science and Technology (Grant Numbers: 20202BABL206053, 20192BAB205045, and 20161BBI90018).

## Conflict of Interest

The authors declare that the research was conducted in the absence of any commercial or financial relationships that could be construed as a potential conflict of interest.

## Publisher's Note

All claims expressed in this article are solely those of the authors and do not necessarily represent those of their affiliated organizations, or those of the publisher, the editors and the reviewers. Any product that may be evaluated in this article, or claim that may be made by its manufacturer, is not guaranteed or endorsed by the publisher.

## Abbreviations

IS: ischemic stroke
ACSC: albumin-corrected serum calcium
COPD: chronic obstructive pulmonary disease
CHD: coronary heart disease.
