# Supplementary material for: Admission Serum Calcium Level and Short-Term Mortality After Acute Ischemic Stroke: A Secondary Analysis Based on a Norwegian Retrospective Cohort
Source: Front Neurol. 2022 Jun 15;13:889518. doi: 10.3389/fneur.2022.889518 (PMC9240666; doi:10.3389/fneur.2022.889518)
Supplement: Supplementary file 1 [file Table_1.DOCX]

Supplementary Material

## Supplementary Tables

**Supplementary Table 1**. Pooled estimates of multivariate regression analysis based on five multiple imputation data

| Exposure | Crude model | Mode Ⅰ | Mode Ⅱ | Model Ⅲ |
| --- | --- | --- | --- | --- |
| ACSC | 2.38 (1.50, 3.78) 0.000235 | 2.50 (1.50, 4.16) 0.000416 | 2.39 (1.40, 4.07) 0.001303 | 2.50(1.45,4.34)  0.001022 |

Data are expressed as OR (95% CI) P-value, ACSC=albumin-corrected serum calcium(mg/dL)

Crude model: not adjusted.

Mode Ⅰ: adjusted for age and gender.

Mode Ⅱ: adjusted for age, gender, serum glucose, atrial fibrillation/atrial flutter, renal insufficiency, heart failure, chronic obstructive pulmonary disease, cancer, pneumonia, paralysis, and aphasia.

Model Ⅲ: adjusted for age, gender, serum glucose, serum-phosphate tertiles, serum-magnesium tertiles, atrial fibrillation/atrial flutter, renal insufficiency, heart failure, chronic obstructive pulmonary disease, cancer, pneumonia, paralysis, aphasia, cognitive disorder, and epilepsy.

**Supplementary Table 2.** Baseline characteristics comparison between included and excluded patients

|  | Included patients | Excluded patients | P-value |
| --- | --- | --- | --- |
| N=886 | 876 | 10 |  |
| Age (years, mean ± SD) | 77.26 ± 12.26 | 83.20 ± 9.51 | 0.127 |
| Gender（male）, n(%) |  |  | 0.737 |
| male | 397 (45.32%) | 4 (40.00%) |  |
| female | 479 (54.68%) | 6 (60.00%) |  |
| Serum-sodium (mmol/L, mean ± SD) | 139.42 ± 3.56 | 139.20 ± 3.85 | 0.845 |
| Serum-glucose (mmol/L, mean ± SD) | 6.91 ± 2.23 | 6.79 ± 1.55 | 0.88 |
| Serum-potassium (mmol/L, mean ± SD) | 4.13 ± 0.43 | 4.11 ± 0.53 | 0.877 |
| Serum-phosphate (mmol/L, mean ± SD) | 1.09 ± 0.29 | 1.05 ± 0.32 | 0.842 |
| Serum-magnesium (mmol/L, mean ± SD) | 0.82 ± 0.09 | 0.72 ± 0.16 | 0.082 |
| Diabetes, n (%) |  |  | 0.334 |
| No | 801 (91.44%) | 10 (100.00%) |  |
| Yes | 75 (8.56%) | 0 (0.00%) |  |
| Hyperlipemia, n (%) |  |  | 0.525 |
| No | 842 (96.12%) | 10 (100.00%) |  |
| Yes | 34 (3.88%) | 0 (0.00%) |  |
| Hypertension, n (%) |  |  | 0.848 |
| No | 637 (72.72%) | 7 (70.00%) |  |
| Yes | 239 (27.28%) | 3 (30.00%) |  |
| Atrial fibrillation/atrial flutter, n (%) |  |  | 0.244 |
| No | 646 (73.74%) | 9 (90.00%) |  |
| Yes | 230 (26.26%) | 1 (10.00%) |  |
| Heart failure, n (%) |  |  | 0.58 |
| No | 850 (97.03%) | 10 (100.00%) |  |
| Yes | 26 (2.97%) | 0 (0.00%) |  |
| Renal insufficiency, n (%) |  |  | 0.417 |
| No | 835 (95.32%) | 9 (90.00%) |  |
| Yes | 40 (4.57%) | 1 (10.00%) |  |
| Chronic obstructive pulmonary disease, n (%) |  |  | 0.709 |
| No | 864 (98.63%) | 10 (100.00%) |  |
| Yes | 12 (1.37%) | 0 (0.00%) |  |
| Coronary heart disease, n (%) |  |  | 0.491 |
| No | 831 (94.86%) | 9 (90.00%) |  |
| Yes | 45 (5.14%) | 1 (10.00%) |  |
| Cancer, n (%) |  |  | 0.657 |
| No | 859 (98.06%) | 10 (100.00%) |  |
| Yes | 17 (1.94%) | 0 (0.00%) |  |
| Malnutrition, n (%) |  |  | 0.638 |
| No | 857 (97.83%) | 10 (100.00%) |  |
| Yes | 19 (2.17%) | 0 (0.00%) |  |
| Dehydration, n (%) |  |  | 0.323 |
| No | 842 (96.12%) | 9 (90.00%) |  |
| Yes | 34 (3.88%) | 1 (10.00%) |  |
| Pneumonia, n (%) |  |  | 0.416 |
| No | 836 (95.66%) | 9 (90.00%) |  |
| Yes | 40 (4.57%) | 1 (10.00%) |  |
| Paralysis, n (%) |  |  | 0.985 |
| No | 790 (90.18%) | 9 (90.00%) |  |
| Yes | 86 (9.82%) | 1 (10.00%) |  |
| Epilepsy, n (%) |  |  | 0.762 |
| No | 868 (99.09%) | 10 (100.00%) |  |
| Yes | 8 (0.91%) | 0 (0.00%) |  |
| Cognitive disorder, n (%) |  |  | 0.566 |
| No | 848 (96.80%) | 10 (100.00%) |  |
| Yes | 28 (3.20%) | 0 (0.00%) |  |
| Aphasia, n (%) |  |  | 0.733 |
| No | 813 (92.81%) | 9 (90.00%) |  |
| Yes | 63 (7.19%) | 1 (10.00%) |  |

**Supplementary Table 3. Effect of magnitude of ACSC on 30-day mortality risk** **stratified by comorbidity and non-comorbidity subgroups**

| Exposure | ACSC (mg/dL) | |
| --- | --- | --- |
| Subgroups | non-comorbidity(n=325) | comorbidity(n=551) |
| Crude Model* | 3.62 (1.51, 8.66) 0.0039 | 1.98 (1.15, 3.40) 0.0141 |
| Mode Ⅰ* | 3.10 (1.22, 7.83) 0.0169 | 2.30 (1.24, 4.25) 0.0078 |
| Mode Ⅱ* | 3.00 (1.07, 8.40) 0.0363 | 2.68 (1.37, 5.23) 0.0038 |

Data are expressed as OR (95% CI) P-value. ACSC=albumin-corrected serum calcium(mg/dL).

Crude model*: not adjusted.

Mode Ⅰ*: adjusted for age and gender.

Mode Ⅱ*: adjusted for age, gender, serum glucose, serum-phosphate tertiles, serum-magnesium tertiles, cognitive disorder, epilepsy, paralysis, and aphasia.

**Supplementary Table 4. Multivariate logistic regression analysis of** **ACSC and 30-day mortality for the patients with secondary diagnosis as ischemic stroke**

| Exposure(n=75) | Crude Model | Mode Ⅰ | Mode Ⅱ |
| --- | --- | --- | --- |
| ACSC | 1.75 (0.49, 6.29) 0.3881 | 1.68 (0.44, 6.47) 0.4476 | 2.15 (0.53, 8.63) 0.2827 |

Data are expressed as OR (95% CI) P-value, ACSC=albumin-corrected serum calcium(mg/dL)

Crude model: not adjusted.

Mode Ⅰ: adjusted for age and gender.

Mode Ⅱ: adjusted for age, gender, serum glucose, atrial fibrillation/atrial flutter, renal insufficiency, heart failure, chronic obstructive pulmonary disease, cancer, pneumonia, paralysis, and aphasia

**Supplementary Table 5. Multivariate regression analysis of ACSC and 7-day mortality.**

| Exposure | Crude model | Mode Ⅰ | Mode Ⅱ | Model Ⅲ |
| --- | --- | --- | --- | --- |
| ACSC | 1.74 (1.00, 3.02) 0.0505 | 1.77 (0.96, 3.26) 0.0693 | 1.61 (0.83, 3.11) 0.1593 | 1.70 (0.85, 3.39) 0.1313 |

Data are expressed as OR (95% CI) P-value, ACSC=albumin-corrected serum calcium(mg/dL)

Crude model: not adjusted.

Mode Ⅰ: adjusted for age and gender.

Mode Ⅱ: adjusted for age, gender, serum glucose, atrial fibrillation/atrial flutter, renal insufficiency, heart failure, chronic obstructive pulmonary disease, cancer, pneumonia, paralysis, and aphasia.

Model Ⅲ: adjusted for age, gender, serum glucose, serum-phosphate tertiles, serum-magnesium tertiles, atrial fibrillation/atrial flutter, renal insufficiency, heart failure, chronic obstructive pulmonary disease, cancer, pneumonia, paralysis, aphasia, cognitive disorder, and epilepsy.

**Supplementary Table 6. The colinear analysis of covariates**

|  | VIF |
| --- | --- |
| ACSC | 1.1 |
| Age | 1.3 |
| Gender | 1.2 |
| Serum-sodium | 1.1 |
| Serum-glucose | 1.4 |
| Serum-potassium | 1.1 |
| Diabetes | 1.4 |
| Cancer | 1 |
| Malnutrition | 1 |
| Hypertension | 1.1 |
| Hyperlipemia | 1.1 |
| Atrial fibrillation/atrial flutter | 1.1 |
| Heart failure | 1.1 |
| Renal insufficiency | 1.1 |
| COPD | 1.1 |
| CHD | 1.1 |
| Paralysis | 1.3 |
| Epilepsy | 1 |
| Cognitive disorder | 1.1 |
| Aphasia | 1.2 |
| Pneumonia | 1.1 |
| Dehydration | 1.1 |
| Serum-phosphate tertiles | 4.1 |
| Serum-magnesium tertiles | 4.1 |

**Supplementary Table 7. The correlations between covariates and 30-day mortality**

| **Covariates** | **N** | **beta** | **Se.** | **exp(beta)** | **95%CI Low** | **95%CI Upp** | **P.value** |
| --- | --- | --- | --- | --- | --- | --- | --- |
| Age | 876 |  |  |  |  |  | <0.0001 |
| Gender | 876 | 0.2951 | 0.2337 | 1.3433 | 0.8496 | 2.1238 | 0.2067 |
| Serum-sodium | 875 | 0.0448 | 0.0346 | 1.0458 | 0.9772 | 1.1193 | 0.1956 |
| Serum-glucose | 876 | 0.1077 | 0.0418 | 1.1137 | 1.026 | 1.2089 | 0.01 |
| Serum-potassium | 873 | 0.3348 | 0.2579 | 1.3976 | 0.843 | 2.317 | 0.1943 |
| Diabetes | 876 | -0.2298 | 0.4418 | 0.7947 | 0.3343 | 1.8893 | 0.603 |
| Cancer | 876 | 0.7083 | 0.6467 | 2.0305 | 0.5716 | 7.2127 | 0.2734 |
| Malnutrition | 876 | 0.0927 | 0.7564 | 1.0971 | 0.2491 | 4.8319 | 0.9025 |
| Hypertension | 876 | -0.618 | 0.2952 | 0.539 | 0.3022 | 0.9615 | 0.0363 |
| Hyperlipemia | 876 | -15.3794 | 678.4793 | 0 | 0 | Inf | 0.9819 |
| Atrial fibrillation/atrial flutter | 876 | 0.4188 | 0.2429 | 1.5201 | 0.9443 | 2.4471 | 0.0847 |
| Heart failure | 876 | 1.8654 | 0.4209 | 6.4583 | 2.8306 | 14.7355 | <0.0001 |
| Renal insufficiency | 876 | 1.3625 | 0.3745 | 3.9059 | 1.8749 | 8.137 | 0.0003 |
| COPD | 876 | 1.1565 | 0.6767 | 3.1789 | 0.8438 | 11.9753 | 0.0874 |
| CHD | 876 | 0.1599 | 0.4887 | 1.1734 | 0.4503 | 3.0581 | 0.7435 |
| Paralysis | 876 | 0.5743 | 0.3254 | 1.7759 | 0.9384 | 3.3606 | 0.0776 |
| Epilepsy | 876 | -13.3456 | 514.5607 | 0 | 0 | Inf | 0.9793 |
| Cognitive disorder | 876 | -1.0881 | 1.0243 | 0.3369 | 0.0452 | 2.508 | 0.2881 |
| Aphasia | 876 | 0.8693 | 0.3435 | 2.3852 | 1.2165 | 4.6764 | 0.0114 |
| Pneumonia | 876 | 2.0029 | 0.3464 | 7.4106 | 3.7586 | 14.611 | <0.0001 |
| Dehydration | 876 | -0.1088 | 0.6157 | 0.8969 | 0.2683 | 2.9982 | 0.8598 |
| Serum-phosphate tertiles | 876 | 0.4731 | 0.4599 | 1.6049 | 0.6516 | 3.9529 | 0.3036 |
|  |  | 0.3669 | 0.4516 | 1.4433 | 0.5956 | 3.4977 | 0.4165 |
|  |  | -0.0652 | 0.3802 | 0.9369 | 0.4447 | 1.9739 | 0.8639 |
| Serum-magnesium tertiles | 876 | 0.0445 | 0.4714 | 1.0455 | 0.415 | 2.6338 | 0.9249 |
|  |  | 0.4881 | 0.4244 | 1.6292 | 0.7091 | 3.743 | 0.2501 |
|  |  | -0.1538 | 0.3656 | 0.8575 | 0.4188 | 1.7555 | 0.674 |

**Supplementary Table 8. The change of regression coefficient of albumin-corrected serum calcium.**

| **Covariates** | **ACSC (regression coefficient)** | **ACSC (regression coefficient)** | **screen out** |
| --- | --- | --- | --- |
|  | 0.8768 | 0.9834 |  |
| Age | 0.8669 | 0.8505 * | Yes |
| Gender | 0.8546 | 0.9558 |  |
| Serum-sodium | 0.8908 | 1.0043 |  |
| Serum-glucose | 0.8594 | 1.038 |  |
| Serum-potassium | 0.8723 | 0.988 |  |
| Diabetes | 0.8808 | 0.9949 |  |
| Cancer | 0.8575 | 1.0634 |  |
| Malnutrition | 0.8823 | 0.9734 |  |
| Hypertension | 0.8723 | 0.9903 |  |
| Hyperlipemia | 0.8916 | 0.9808 |  |
| Atrial fibrillation/atrial flutter | 0.8705 | 0.9852 |  |
| Heart failure | 0.8827 | 0.9897 |  |
| Renal insufficiency | 0.88 | 1.0005 |  |
| COPD | 0.8937 | 0.9619 |  |
| CHD | 0.8814 | 0.9831 |  |
| Paralysis | 0.8649 | 0.984 |  |
| Epilepsy | 0.8715 | 0.9866 |  |
| Cognitive disorder | 0.9036 | 0.9493 |  |
| Aphasia | 0.8833 | 0.9778 |  |
| Pneumonia | 0.8618 | 1.0242 |  |
| Dehydration | 0.88 | 0.9908 |  |
| Serum-phosphate tertiles | 0.8737 | 0.8903 |  |
| Serum-magnesium tertiles | 0.8813 | 0.9118 |  |

**Supplementary Table 9. Comparison with the other clinical studies**

| **Year** | **Journal** | **population** | **outcome** | **results** |
| --- | --- | --- | --- | --- |
| 2007 | Arch Neurol | American | infarct volumes | negative correlation |
| 2008 | Stroke | American | 3-month functional outcome | negative correlation |
| 2010 | Cerebrovasc Dis | [Israel](javascript:;) | long-term mortality | non-linear correlation |
| 2015 | J Stroke | Korean | long-term mortality | positive correlation |
| 2017 | JPakMed Assoc | Japan | admission NIHSS scores | negative correlation |
| 2021 | Atherosclerosis | Chinese | long-term mortality | positive correlation |
| 2022 | Our study | Norwegian | short-term mortality | positive correlation, linear relationship |

**Supplementary Table 10. Multivariate logistic regression analysis of ACSC and 1-year mortality**

| Exposure | Crude model | Mode Ⅰ | Mode Ⅱ | Model Ⅲ |
| --- | --- | --- | --- | --- |
| ACSC(continuous) | 2.16 (1.48, 3.14) | 2.19 (1.46, 3.28) | 2.08 (1.37, 3.15) | 2.29 (1.48, 3.55) |

Data are expressed as OR (95% CI) P-value. ACSC=albumin-corrected serum calcium(mg/dL).

Crude model: not adjusted.

Mode Ⅰ: adjusted for age and gender.

Mode Ⅱ: adjusted for age, gender, serum glucose, atrial fibrillation/atrial flutter, renal insufficiency, heart failure, chronic obstructive pulmonary disease, cancer, pneumonia, paralysis, and aphasia.

Model Ⅲ: adjusted for age, gender, serum glucose, serum-phosphate tertiles, serum-magnesium tertiles, atrial fibrillation/atrial flutter, renal insufficiency, heart failure, chronic obstructive pulmonary disease, cancer, pneumonia, paralysis, aphasia, cognitive disorder, and epilepsy.

## Figures

**Supplementary Figure 1.** Curve fitting of five different models

**
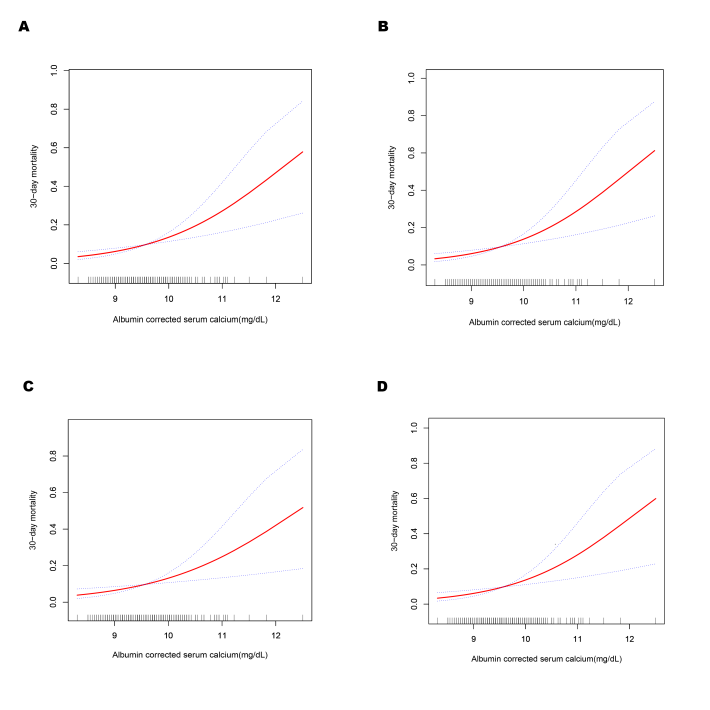
**

Data are expressed as OR (95% CI) P-value, ACSC=albumin-corrected serum calcium(mg/dL)

A-Crude model: not adjusted.

B-Mode Ⅰ: adjusted for age and gender.

C-Mode Ⅱ: adjusted for age, gender, serum glucose, atrial fibrillation/atrial flutter, renal insufficiency, heart failure, chronic obstructive pulmonary disease, cancer, pneumonia, paralysis, and aphasia.

D-Model Ⅲ: adjusted for age, gender, serum glucose, serum-phosphate tertiles, serum-magnesium tertiles, atrial fibrillation/atrial flutter, renal insufficiency, heart failure, chronic obstructive pulmonary disease, cancer, pneumonia, paralysis, aphasia, cognitive disorder, and epilepsy.

The red line represents the best-fit line, and the blue lines are 95% confidence intervals. The potential demarcation point was 9.6mg/dL according to the smoothing spline plots.

**Supplemental Figure 2.** The process of multiple imputation

**
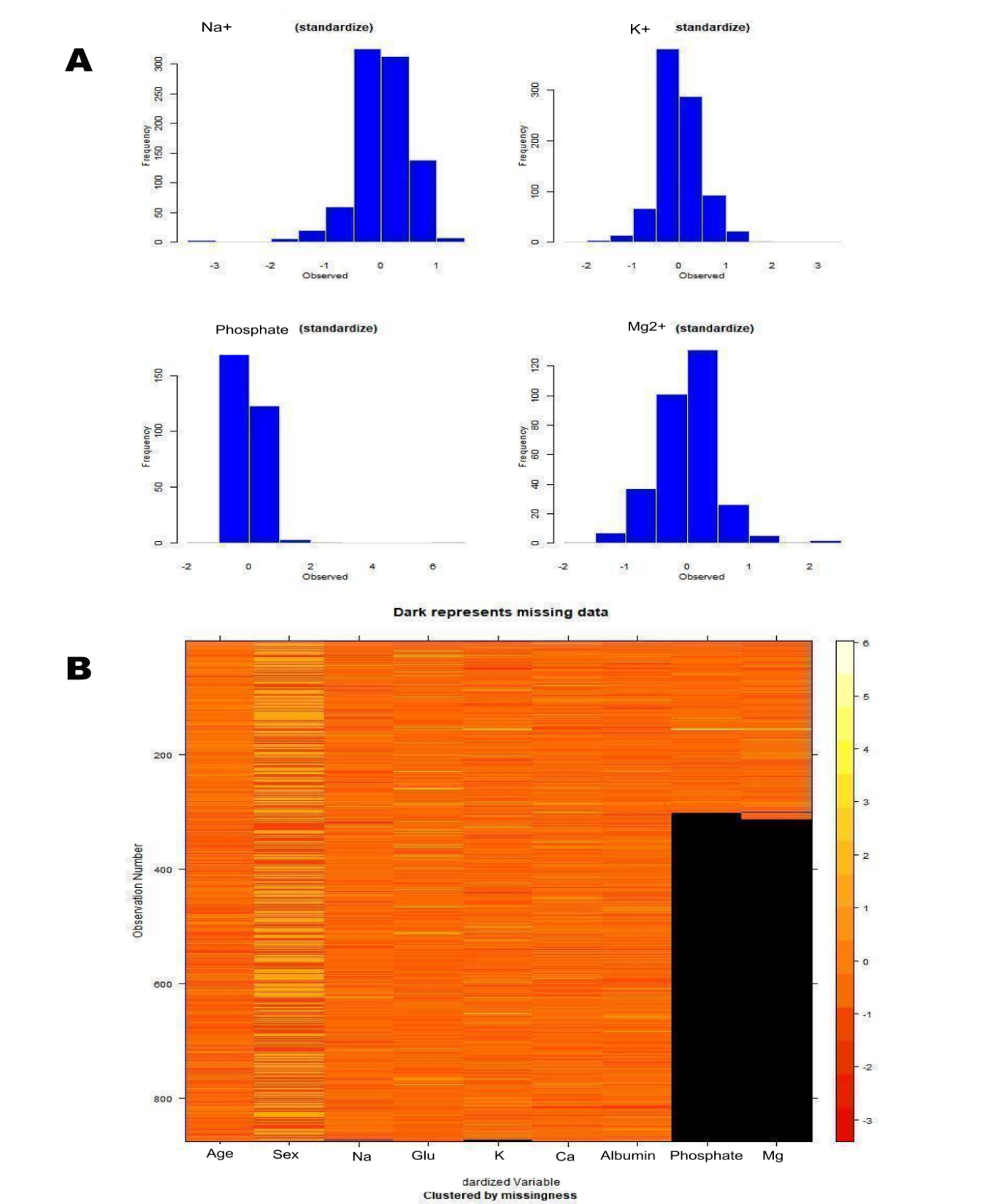
**

**Supplementary Figure** **3**. ROC curve of blood ions for 30-day mortality

**
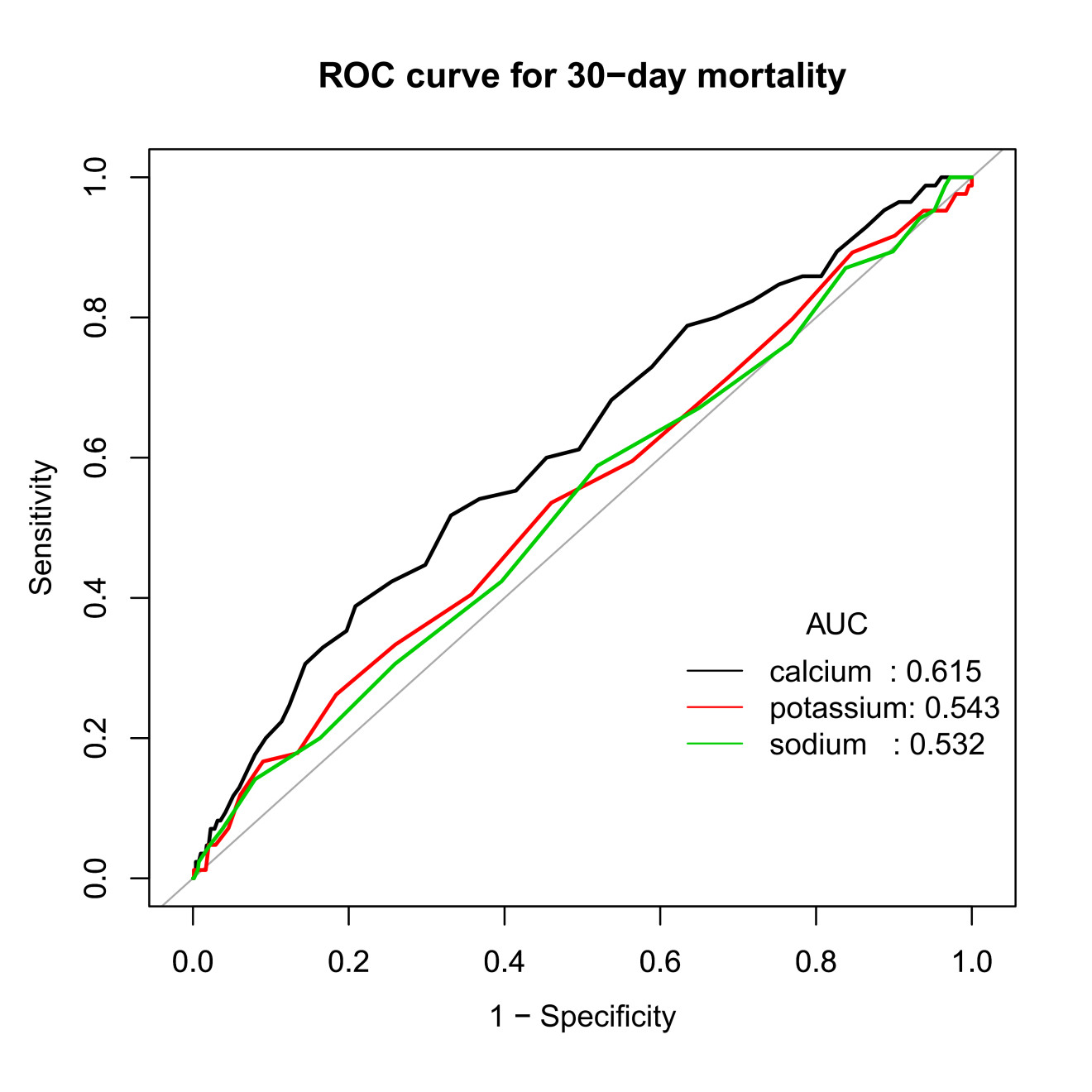
**
